# Towards Recognition of Human Actions in Collaborative Tasks with Robots: Extending Action Recognition with Tool Recognition Methods

**DOI:** 10.3390/s23125718

**Published:** 2023-06-19

**Authors:** Lukas Büsch, Julian Koch, Daniel Schoepflin, Michelle Schulze, Thorsten Schüppstuhl

**Affiliations:** Hamburg University of Technology, Institute of Aircraft Production Technology, Denickestraße 17, 21073 Hamburg, Germany

**Keywords:** assembly, tool detection, tool recognition, human action recognition, industrial object recognition, assembly step recognition, assembly progress detection, human–robot collaboration

## Abstract

This paper presents a novel method for online tool recognition in manual assembly processes. The goal was to develop and implement a method that can be integrated with existing Human Action Recognition (HAR) methods in collaborative tasks. We examined the state-of-the-art for progress detection in manual assembly via HAR-based methods, as well as visual tool-recognition approaches. A novel online tool-recognition pipeline for handheld tools is introduced, utilizing a two-stage approach. First, a Region Of Interest (ROI) was extracted by determining the wrist position using skeletal data. Afterward, this ROI was cropped, and the tool located within this ROI was classified. This pipeline enabled several algorithms for object recognition and demonstrated the generalizability of our approach. An extensive training dataset for tool-recognition purposes is presented, which was evaluated with two image-classification approaches. An offline pipeline evaluation was performed with twelve tool classes. Additionally, various online tests were conducted covering different aspects of this vision application, such as two assembly scenarios, unknown instances of known classes, as well as challenging backgrounds. The introduced pipeline was competitive with other approaches regarding prediction accuracy, robustness, diversity, extendability/flexibility, and online capability.

## 1. Introduction

Efficient Human–Machine Interfaces (HMIs) are the communication basis between humans and technical systems, enabling sophisticated applications arising within Industry 4.0. Especially in Human–Robot Collaboration (HRC), where the mutual awareness of the worker and the system is crucial for a fluent workflow, efficient HMIs are required [1,2]. In this context, well-designed HMIs are the basis for a natural, safe, and effective collaboration in a shared workspace [3]. These interfaces are supposed to transfer information from the machine to the human worker and vice versa. Here, the communication channel from the machine to the human worker primarily serves the purposes of safety. Furthermore, these interfaces can increase the human worker’s acceptance of the machine, enabling collaboration by facilitating the coordination of tasks between the human and the robotic system. Several technical solutions are available to realize this communication channel. Examples are visual systems (e.g., to display texts or videos) or haptic systems (e.g., to trigger vibration alerts). Moreover, acoustic signals can be used, such as speech output and other sound signals [4]. On the other hand, the communication channel from the human to the machine should enable the consideration of the human’s actions by the machine. This is required, among others, for proper coordination of tasks and safety issues [5]. Especially for the coordination of tasks, it is essential to be able to detect the progress in the process. Various technical solutions are available for this, e.g., mechanical feedback, such as simple push buttons, or verbal feedback, such as voice input. However, these feedback mechanisms are based on non-value-adding activities for the human worker, which can disrupt the workflow of the whole process. Depending on the respective field of application, the design of HMIs is challenging in different ways. For example, the demands placed on HMIs can become more complex as the need for process flexibility increases, as is the case with the assembly of multi-variant products.

### 1.1. Application Domain

Flexible manufacturing processes of multi-variant products in human–robot collaboration are mainly found in small batch production, for example in the production of aircraft interiors [6]. However, examples can also be found in other application areas, such as the small batch production of hydrogen electrolyzers [7]. Across application domains, these types of processes require automated progress detection capable of capturing small step process progress in the assembly of multi-variant products. This provides the foundation for efficient orchestration systems and enables efficient HRC assembly systems. Therefore, a generic HRC assembly process of a small batch production involving a worker, a cobot, and hand tools was assumed as a use case, forming the basis for subsequent investigations.

### 1.2. Research Gap

Facing the issues of established human–machine interfaces, approaches, and strategies to acquire information without active human involvement has emerged within the manufacturing domain. Various methods either observe the workpiece, the tools, or the human in the process to derive the assembly progress [8,9,10]. Thereby, as will be argued in Section 2, the methods of Human Action Recognition (HAR) [11,12] are of particular relevance to this work. HAR feedback methods in manufacturing focus on the movements and actions of the human worker to obtain information about the current assembly process for inferring the overall assembly progress. For this, the human pose and movements are tracked by localization and motion-capturing systems such as camera systems or inertial measurement units. These HAR methods have several advantages in application to flexible manufacturing systems and handling multi-variant products without introducing non-value-adding process steps for the human worker [11,13]. However, current implementations of HAR methods for automated progress detection in multi-variant assembly processes show deficits. Among others, one large deficit of such HAR-based systems is the lack of the accuracy and reliability of the results.

To improve the accuracy and reliability of HAR-based assembly-progress-detection systems, one approach is to acquire additional contextual information about the assembly process. Besides the human movements and actions, the tools used by the human worker also provide insights into the performed assembly steps [9], as the solution space of possible assembly steps can be reduced when the current tool in use is known.

Although the research area of tool recognition, which belongs to the field of object recognition, is of high interest, there are still some unresolved challenges in making object recognition applicable to HAR in the use case presented above. A holistic approach that enables tool recognition for manual assembly processes is missing in the application field of assembly progress recognition. However, there are isolated approaches that serve as partial solutions to the problem and indicate that the principle is plausibly applicable to the underlying use case. Therefore, this work contributes to the development and implementation of a tool-recognition method in manual assembly processes that can be integrated into existing HAR-based methods for progress detection. For this purpose, the interim objectives were defined as the investigation of necessary tool-recognition modules and the development and presentation of a novel framework for tool recognition that can be integrated into existing HAR systems. Thereby, the solution was developed, evaluated, and classified in the state of research based on the following aspects: prediction accuracy, robustness, diversity, extendability/flexibility, and online capability.

### 1.3. Outline of This Work

**Figure 1:**
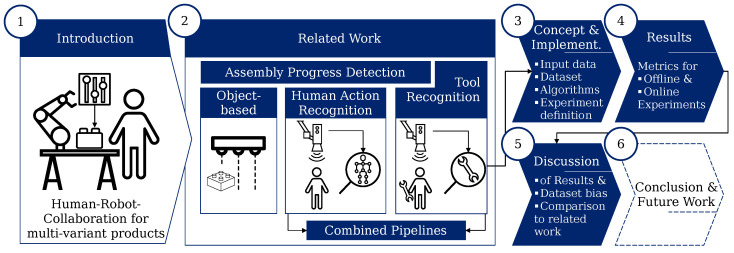
Structure and main contents of this work. The sections are marked with numbers in circles (Section 1, Section 2, Section 3, Section 4, Section 5 and Section 6).

The paper first examined the state of research (Section 2) from progress detection in manual assembly via HAR-based methods to visual tool recognition. Then, a novel concept for tool recognition to assist HAR-based assembly-progress-estimation methods was developed and implemented. Therefore, an implementation of the method in software and hardware was conducted, and a training dataset was created. To test the given approach with the implemented classification algorithms, a set of offline and online experiments was defined as well (Section 3). In the following section, first, the implemented classification algorithms were optimized using a hyperparameter study. Then, the defined experiments were performed and the results presented in the form of performance metrics (Section 4). In the following, the results of the offline classification, as well as the online experiments are discussed, with special emphasis on the achieved accuracies, frame rates, and dataset bias. Based on this, the current state of development was compared with the presented papers of the Related Work Section, and the impact on assembly step estimation is explained (Section 5). Finally, the paper is summarized and different potentials for future work are highlighted in Section 6. The structure and main contents of this work are depicted in Figure 1.

## 2. Related Work

In this section, the state of research on progress detection in manual assembly is presented and discussed. For this purpose, the topics of Assembly Step Estimation (ASE) and Human Action Recognition (HAR) are addressed. Furthermore, the state of research on visual tool recognition is discussed. Recent work on tool-recognition methods, as well as issues of data availability are discussed herein.

### 2.1. Progress Detection in Manual Assembly

Three steps are required to automatically detect the progress of a manual assembly: data acquisition, data preparation, and data evaluation [14]. Data acquisition describes the interface between the physical process and the computer system that performs progress detection. Several different methods to acquire feedback from the manual assembly process are available in the state of research and development [3]. In this regard, these methods can be categorized as manual and automatic feedback methods. Whereas manual feedback methods are accompanied by introducing non-value-adding tasks into the workflow—such as pressing buttons or giving haptic, acoustic, or visual feedback to confirm completed assembly steps—on the other hand, automatic feedback methods gain the necessary information by observing the process without introducing feedback steps into the assembly plan. The observation of the assembly process can be conducted by optical [15] or gyroscopic [16] systems that track the position or movement of the worker, tools, and other objects in the workspace. Furthermore, smart tools can deliver meaningful information by transmitting their operation status and other collected data.

#### 2.1.1. Assembly Step Estimation

Subsequent to the data acquisition, the collected data need to be processed to obtain a proper estimation of the assembly progress. For this, the vast majority of available methods from the state of research identify the current assembly step based on the acquired data and subsequently estimate the overall assembly progress respecting the assembly plan and the history of detected assembly steps [11].

The necessary information about the assembly progress can be obtained by **observing the product** during the process [8,17]. The variation of the product throughout the process is a meaningful parameter to determine the progress of the process. However, this is not always applicable if the product is multi-variant with small lot sizes and, as a result, the data availability is poor or if the assembly situation is chaotic and affected by occlusions. Therefore, observing the product to determine the assembly progress was not considered in the following.

A generic, process-independent method to detect assembly steps is presented in [9]. Here, an **object detection** system is trained to detect handheld tools in use by the human worker to derive assembly steps from the tool usage. To this end, additional optical pose estimation is applied to determine the usage times of the tools by the worker. The information about the tools in use is subsequently used to estimate the current assembly step. Although this generic ASE method can be applied for cross-process usage and multi-variant processes, its application is limited since the method can only distinguish between assembly steps with different tools in use and is, therefore, not necessarily small step applicable.

Besides the above-mentioned methods, **HAR-based** ASE methods have recently been gaining scientific relevance. For HAR, the poses and movements of humans in the process are captured by localization and motion-capturing systems such as camera systems or inertial measurement units. Based on this information, their current assembly action is inferred [11,15]. In [18,19], a Hidden Markov Model (HMM) is used to learn the trajectories of the human worker during the assembly process. The HMM is trained on task-specific trajectories conducted by the worker, which can then be recognized during task execution. However, the learned trajectories are task- and subject-specific and are, therefore, not applicable to cross-process usage and multi-variant processes since they are trained on specific processes in specific work environments. Another approach represents the assembly process with assembly states in a Finite-State Machine (FSM) [10]. Using video-based object detection and localization, state transitions — and thereby the assembly progress—are detected. Although this method is well comprehensible, the size of the FSM scales linearly with the number of assembly steps in the assembly plan. Thus, this method is accompanied by a large implementation effort, which makes this method not economical for long assembly tasks and small lot sizes. Furthermore, this method is also not applicable to multi-variant products and their processes without generating new task models for each variant.

In order to keep the HAR generic and, thus, to ensure the applicability to multi-variant processes, process-independent basic actions are utilized, with which arbitrary processes can be modeled [11,13,20]. In the assembly context, basic human actions for process description are an established tool [21]. In Methods-Time Measurement (MTM) analysis, basic actions with different levels of detail are used to describe and predict manual assembly processes. These MTM primitives contain the means of trajectories and execution times of workers with varying levels of skill. Several publications use MTM primitives as basic actions for process descriptions, which have to be recognized for ASE [11,13].

Since HAR for ASE is a highly frequented field of research, several methods are available. However, recent studies reveal a lack of accuracy and poor performance of the Neural Network approaches [11,16]. Furthermore, the depicted action classes do not adequately represent complex assembly processes accompanied by a lack of suitable training data [22,23]. In summary, besides some open deficits, the state of research offers promising solutions for automatic progress detection in manual and multi-variant assembly. A promising modular concept for ASE was developed in [11]. Despite some deficits in the implementation of ASE, a promising approach to integrate tool recognition to improve the accuracy of ASE was proposed, but not further developed and implemented. Therefore, this work contributes to the investigation of the tool-recognition module proposed in the concept of [11].

#### 2.1.2. Human Action Recognition Pipeline

In the following, the pipeline of the developed HAR ASE method in our previous work [11] that enables the integration of optical tool recognition is exemplarily described. The investigation of the used pipeline demonstrates the incorporation of a tool-recognition system into an existing HAR method. Nonetheless, the methodology developed in this paper also provides scientific value to other HAR methodologies, as its integration is not limited to our HAR methodology.

**Figure 2:**
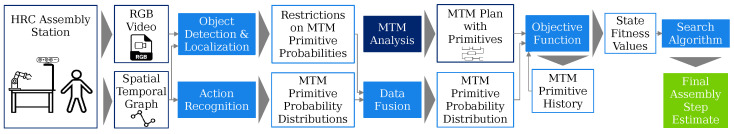
Concept for assembly step estimation: A methods-time-measurement-based approach [11].

In [11], the workspace is observed with an Azure Kinect [15], which captures RGB video and skeleton data. The skeleton data are fed through a Neural Network [24] trained to recognize MTM primitives. These MTM primitives correspond to certain assembly steps. In addition to the MTM primitive recognition, the RGB video and the skeleton data are fed to a tool-detection system. Detecting the use of handheld tools can provide important insights by adding additional constraints to the estimated assembly step [9]. Fusing the obtained data under the consideration of the detection history and the assembly plan, the assembly progress is estimated. The concept developed in [11] is depicted in Figure 2.

### 2.2. Visual Tool Recognition

First, a detailed analysis of existing approaches in the field of tool recognition in manual assembly situations is reviewed and discussed based on evaluation criteria. The section concludes with an analysis of available datasets and their suitability for the discussed problem domain.

#### 2.2.1. Tool-Recognition Work

To review the literature and assess the suitability of existing approaches toward the above-introduced framework, five evaluation criteria are introduced:

1. **Prediction accuracy**: Sufficient tool prediction accuracy is necessary to avoid misclassifications of detected tools.
2. **Robustness**: Robustness of the tool-recognition applications is necessary to account for changes in the application environment, such as backgrounds and lighting.
3. **Tool/object/action diversity**: Diversity of the tool spectrum considered.
4. **Extendability and flexibility**: If new components/processes/tools are introduced, extensions of the tool-recognition applications are necessary. This criterion evaluates whether the considered approach can be extended with new classes and how it handles non-tool-related manual processes.
5. **Online capability**: Capability of the approach to perform at 30 FPS as it is to be used in near real-time environments.

Previous work in this field focused on the visual recognition/identification of manually performed assembly steps and/or handled tools [9,25,26]. They have shown the principal applicability of such visual approaches for the considered task. This is of particular importance since tools that are handled manually may be heavily occluded and visual identification can be considered challenging.

The above criteria may not be fully applicable to all considered approaches. This is due to the interaction between some of the criteria such as robustness and accuracy. For example, a highly accurate approach to tool recognition may not have been tested in situations where its robustness was evaluated. It can be assumed that achieving high accuracy becomes more challenging as the level of robustness increases. Similar behavior can be observed with the criteria of accuracy and diversity, as high identification complexity generally leads to reduced accuracies. Nonetheless, the differentiation between the approaches investigated under the consideration of those aspects offers a detailed analysis of their respective capabilities. The following attribution of points in each criterion for the methods from the state of research is a comparison of the different approaches and is justified in the following paragraphs. Especially, the prediction accuracy and robustness of the architectures applied to different training datasets are difficult to quantify. This is due to unequal boundary conditions (e.g., varying class quantities, physical setups, input data quality), and therefore, an estimation of the applicability of the underlying use case as made based on the findings in the corresponding papers.

In [25], a manual screw-tightening operation in the automotive domain is considered. A multi-sensor setup is applied, consisting of radio and optical sensors for positional localization and IMU sensors for motion sensing. The latter one is utilized as an attachable module with which the different considered screwing operations are contextually classified. The optical sensors are utilized with markers to localize the screwdriver and associate it with a certain operation. Although the optical localization accuracy is not discussed in detail, the overall results imply sufficient accuracy for its application in manual assembly. Due to the multi-sensor setup, as well as marker-based optical tracking, the robustness is considered high. However, the approach lacks tool diversity and extendability as only a single tool is considered and additional markers have to be attached to the tools. Non-tool-related processes cannot be captured by the approach. Online capability is not specifically mentioned, but can be inferred as sufficient for the considered use case introduced in Section 1.1. The degree of automation is high since no additional actions have to be undertaken by the user.

In comparison to [25], Ref. [9] focuses on a purely AI-based vision analysis to perform an assembly action recognition. They utilize an object detector to detect tools in images and combine this tool-identification model with a 2D and 3D human-pose-estimation model. Both outputs are used to predict one of three actions, wrenching, filing, and hammering. YOLO v3 was used as an object detector and trained with a self-captured dataset. Over the three classes, the identification accuracy is stated to be 92.8% for the tool identification and up to 88.9% for the pose estimation approach. Chen et al. [9] challenged the robustness of their approach by adding up to 10% artificial salt-and-pepper-noise. The approach fails at 10% noise. However, the training and test sets do not vary with respect to light or different backgrounds. Thus, this approach most likely is not transferable to new scenarios, different users, and more complex contexts. Additionally, the three tool classes offer little transferability to complex real-world assembly scenarios. The online capability of the approach yields 33.3 FPS for the YOLOv3 implementation, which is a sufficient frame rate for a possible near real-time application.

Similar to the cascading approach from [9], Ref. [26] utilizes a cascade of YOLOv4 networks to classify defective objects in manual assembly situations. Although not a tool-recognition approach, the shown cascade offers the opportunity to extract relevant sections of the image. This type of two-stage analysis yields significantly improved results in comparison to the one-stage approach.

**Figure 3:**
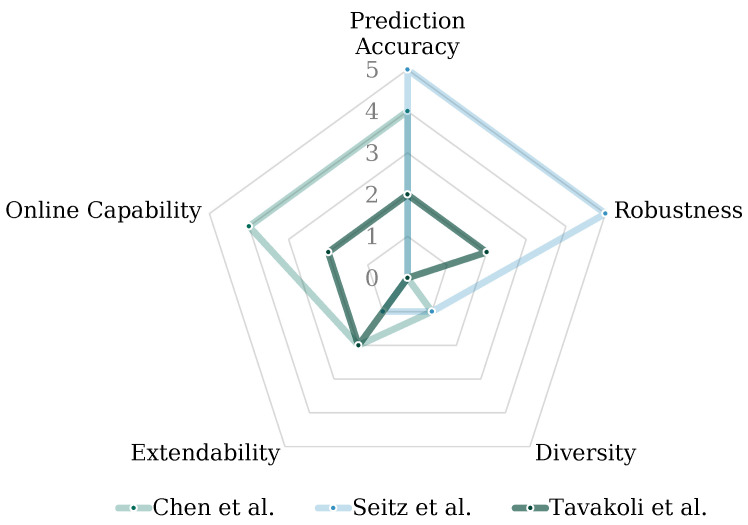
Spider chart representation of the three approaches [9,25,26] that perform visual tool recognition. Each of these approaches was evaluated with respect to the five above-introduced criteria.

The summarized review of existing literature according to the above-defined criteria is visualized in Figure 3. The principle of tool recognition is applicable and yields sufficient accuracy. However, combining online capability with tool diversity is yet unsolved. Furthermore, the existing concepts are little extendable towards new components, processes, or tools. Thus, this paper presents a framework that can be extended while achieving a high tool diversity and online capability while maintaining the high prediction accuracies and robustness features of the reviewed approaches.

#### 2.2.2. Data Availability for Tool Recognition

With the emergence of data-based visual applications, the necessity for sufficient training data leads to several public datasets. Out of the datasets that represent common objects, ImageNet [27] includes data for five tools, MS Coco [28] for two tools, and OpenImage [29] for 13 tool-related classes.

The WorkingHands dataset [30] contains 13 classes of common tools, such as erasers, scissors, pencils, and similar tools. Each class is represented with several hundred annotation instances. Although the WorkingHands dataset contains a large number of images for the instances, the transferability towards industrial use cases is limited with missing tool classes such as files, riveters, soldering irons, etc.

Specialized in equipment and tools, the ALET dataset [31] represents 49 classes of safety equipment and manual/electric tools. Common objects were data-crawled, and specialized objects were generated synthetically. Although this dataset contains a comparatively large amount of classes, the instances per class are imbalanced. Several classes contain less than 200 images compared with above 1000 from other classes. Such imbalance may challenge the aforementioned accuracy goals.

Therefore, in order to enable a holistic and generalizable tool-recognition toolbox, it is necessary to make additions to these datasets. Thus, this paper considered adding new classes to existing datasets and extending instances of existing classes.

## 3. Tool-Recognition Pipeline—Concept and Implementation

**Figure 4:**
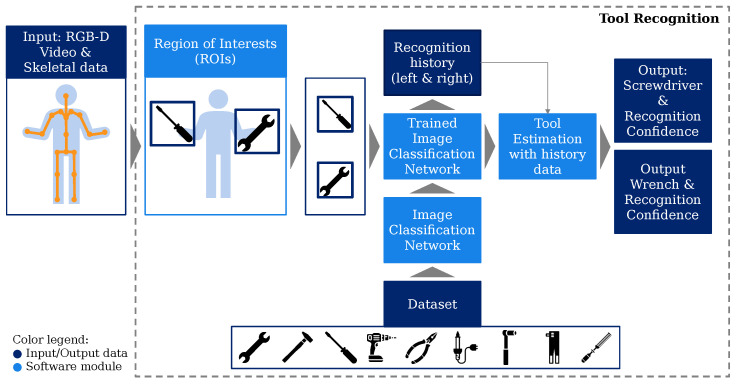
Schematic representation of the concept for tool recognition.

Tool recognition as a component of the entire ASE pipeline is conceptually divided into individual steps, which are shown in Figure 4. In this section, first, the general concept is described, and then, one possible implementation of the modules, which is used for later experiments, is specified in subsections.

The input data required for the approach consisted of the RGB-D video stream and the skeletal data of the employee performing the process. We propose to calculate the skeletal data via the video stream instead of IMU sensors on the employee since this method is robust against drift and, at the same time, non-intrusive. This way, no effort is required for commissioning, i.e., applying the sensors to the employee, which also positively influences the acceptance of such a solution. Furthermore, video data are needed for the higher-level concept of HAR in any case, which can further be leveraged for the tool-recognition module. Regardless of the data acquisition method chosen for skeleton data, it commonly consists of a reduced number of artificial joints, which through their linkage (spatial representation), constitute the human skeleton. Each joint has a position and orientation that can change over time (temporal representation) due to human movement [32].

Since this work focused on tools used in manual assembly processes, the hand regions were particularly relevant. Therefore, as the first processing step of tool recognition, the determination of these hand regions as Regions Of Interest (ROIs) is proposed. This approach offers the advantage that the video is reduced to relevant areas, thus minimizing disturbing influences for the subsequent classification module. The primary disturbing influence here is understood to be tools at the workstation, which are not used for the current process, but can still be captured via the coverage area of the camera. For the determination of the ROIs, the wrist nodes from the skeleton data were used, which formed the center point of a square segment of the video for each hand. The wrist was used as a reference point because previous experiments we conducted indicated that the positions of the skeletal data of the hands and fingers are susceptible to interference, whereas the wrist positions can be determined more accurately and stably. Detailed experiments on this, allowing similar conclusions, can be found in [33]. Here, however, only the joints of the hands and wrists were examined; experiments on finger joints were not performed. Furthermore, due to the increased occlusion of the hands by holding a tool, these were not continuously usable and were, therefore, not considered further for our use case.

The individual ROIs of the video sequence were further processed by an image-classification algorithm. The output of the algorithm—per ROI—was supposed to be a class and a classification confidence, which was subsequently recorded within recognition histories. The recognition histories contained the results of a fixed number of previous classification results. The purpose of these histories was to render the subsequent tool estimation more robust against short-term misclassifications, assuming that a tool change cannot occur within a very short time, e.g., in milliseconds. Finally, the tool-estimation module combined the current and history classifications to compute the classes for the tools in use, as exemplified in Figure 4 with the outputs wrench and screwdriver. The goal was to reduce, thereby, the complexity of the image-processing task of the tool-recognition module.

### 3.1. Acquisition of Input Data

**Figure A1:**
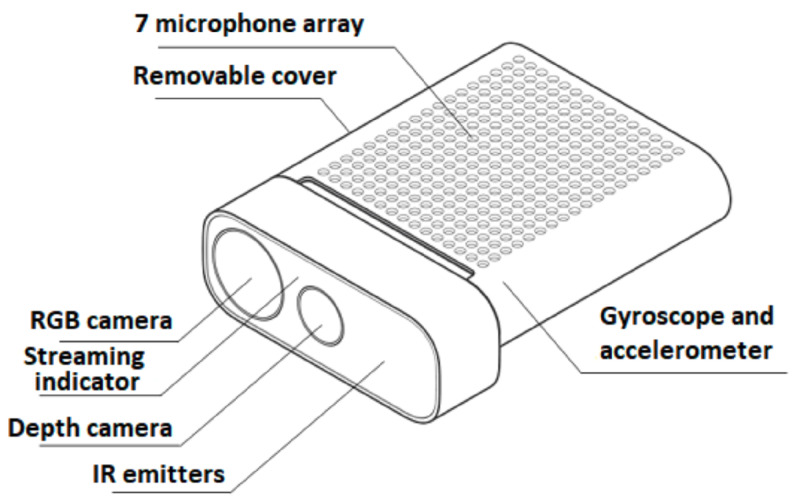
Schematic of the Azure Kinect [34].

**Figure A2:**
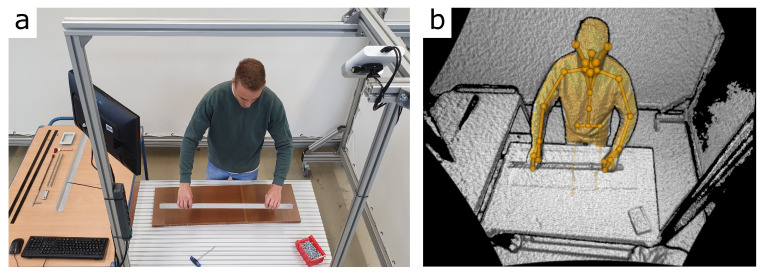
(**a**) Workstation equipped with Azure Kinect. (**b**) View of Azure Kinect [11].

A skeletal representation of human movements acquired by a visual system, as mentioned above, can be obtained with Microsoft’s Azure Kinect [34]. The Azure Kinect, with its software packages, is a state-of-the-art optical body tracking system for research purposes [34,35]. It is equipped with an RGB and an infrared depth camera. The associated software enables skeletal data acquisition based on both RGB and depth image data. Besides the spatial graph representing the skeletal data, the infrared and the RGB video stream are also accessible simultaneously through the software. The skeleton data acquired by the Azure Kinect consist of 32 body joints. These joints include the head, neck, shoulders, elbows, wrists, hands, spine, hips, knees, and ankles, as well as the tips of the fingers and the toes. The schematic representation of the device is depicted in Figure A1, and a possible integration into a workstation and the skeletal data are depicted in Figure A2.

### 3.2. Region of Interest

**Figure 5:**
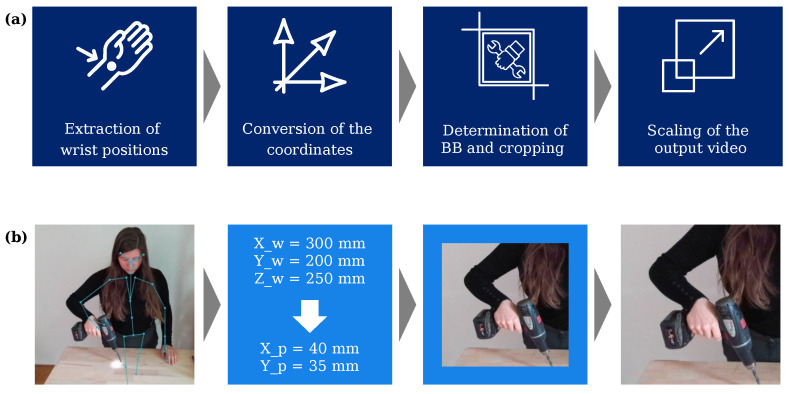
(**a**) Process steps to determine the ROIs; (**b**) application example.

**Figure A3:**
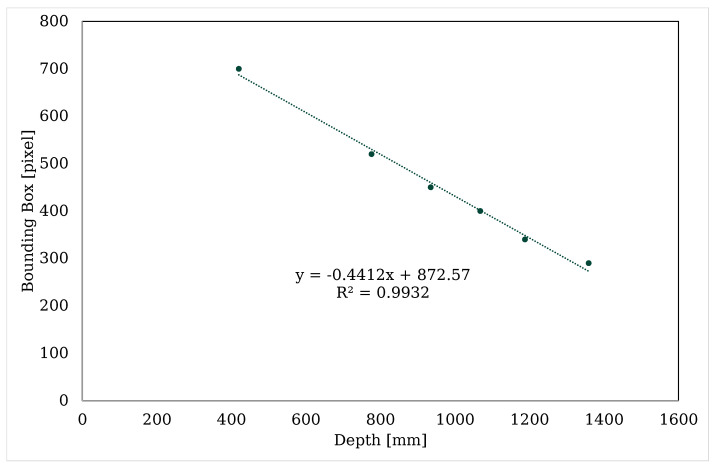
Correlation between depth and bounding box.

The implementation approach for the ROIs was divided into four steps, which are shown schematically (a), as well as exemplary (b) in Figure 5. To implement the ROIs’ determination, we leveraged the Azure Kinect’s skeletal data described in the previous subsection. By utilizing a Python interface [36] to the camera along with the functionalities of the Software Development Kit (SDK), the positions of the wrists were extracted from the skeletal data. The x, y, and z coordinates for each wrist were determined in the coordinate system of the depth camera. In order to extract the Bounding Box (BB) of the video, the coordinates had to be transformed into the coordinate system of the RGB camera. Leveraging the existing transformation functions of the SDK, the 3D coordinates of the depth camera, which were determined in mm, were converted into the 2D pixel coordinates of the RGB camera. The BB was then defined as a square using the pixel coordinates of the wrists as the center point [37]. Since the used tool’s position changes in relation to the camera, the size of the bounding box needs to be adjusted according to the distance from the tool to the camera. For this purpose, the correlation between the BB size and the distance of the tool from the camera needed to be determined. In general, the closer the tool is to the camera, the larger the section of the image must be to ensure that the tool is within the section. To obtain this relation, the largest tool of the dataset (in this work, “drilling machine”) was utilized, and images were taken at different distances from the camera. In addition to the image data, the wrist coordinate was recorded. In the post-processing, a BB for the tool was manually added to the images. This results defined the approximate linear dependence between the bounding box size and the distance of the tool as shown in Figure A3.

This linear relationship was then exploited to crop the video. Using the functions of the OpenCV Library [38], the video was transformed into two videos, each showing the ROI of the respective wrists. Afterward, the video sections were scaled to achieve a uniform size (in this work: 224 × 224 pixels) of the videos. This was necessary because the image classification algorithm uses a uniform input size.

### 3.3. Image Classification

Subsequent to acquisition and preparation, the data needed to be evaluated to classify the tools in the extracted ROI. An image-classification algorithm is proposed for this purpose, raising two key issues: selecting an eligible classification algorithm and the availability of suitable training data. Hence, the dataset and the used classification algorithm are discussed subsequently.

#### 3.3.1. Dataset

To address the data availability problem in the tool-recognition domain discussed in Section 2.2.2, we followed an approach based on combining different data sources. Our image classification dataset was composed of three sources:

- Extracted images from the ALET dataset [31].
- Scraped images from popular search engines, in our case Bing Images (bing.com/images) and Google Images (images.google.com).
- Newly captured photos of real tools held in the hand.

The use of various image sources can enhance the tool variance and expand the size of the dataset. A higher tool variance is beneficial as it increases the robustness of an approach for tools with distinctive characteristics, such as pliers. Additionally, a more extensive dataset aids the classification algorithm in learning from a wide range of examples, which may enhance its performance. Therefore, combining multiple image sources can effectively increase both the quantity and diversity of the dataset.

**Figure 6:**
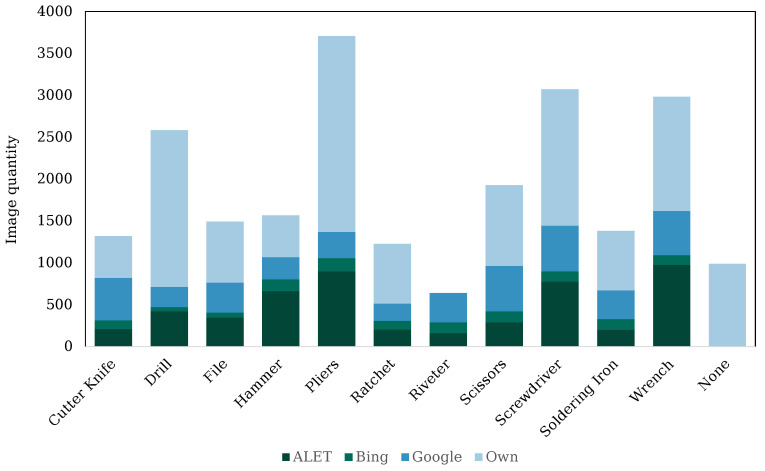
Complete dataset: defined tool classes with shares of the individual data sources.

Before the actual data acquisition, a list of tools relevant to manufacturing was established, which represents the later categories of image classification. The basis for the selection of this tool portfolio was typical tools in the aerospace and automotive industry, as well as the tool classes used in datasets such as ALET [31] or Working Hands [30]. An overall view of the classes used, as well as the distribution among the previously described data sources is shown in Figure 6. In total, there were 12 classes in the dataset, 11 tool classes and an additional “none” class, which contained footage without tool usage. The “none” class is especially important for the application of the developed approach in combination with the HAR approach.

**Figure A4:**
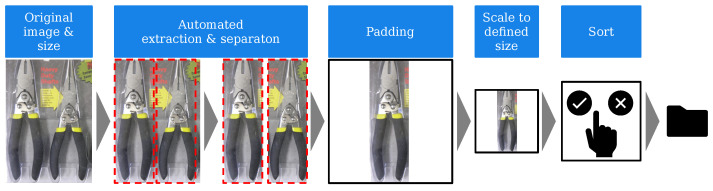
Data acquisition from ALET dataset.

In the following, the data acquisition methods for the different sources are explained. The first source for the dataset was the **ALET** dataset with 49 classes showing objects from tools, safety equipment, and office supplies. Since this dataset was created for object detection, the data must be adapted for the image-classification approach in our work. For this purpose, the individual tools were extracted from the images using the steps shown in Figure A4. The JSON files of the ALET dataset with the information about the number, type, and position of objects within an image were utilized to enable a script that extracted the individual relevant image sections of the respective image of the dataset. After extraction, the individual images were padded to a square format, i.e., the image was enlarged with fill data (white background). Since the image classification method requires a uniform image size, the padded image sections were scaled to the defined size of 224 × 224 pixels (see Section 3.2). Subsequently, the resulting image was saved within its defined class. Additionally, the images were then manually screened; wrongly sorted images were re-sorted; images of inferior quality (resolution) were sorted out. Furthermore, images on which no tool could be recognized were removed. This procedure resulted in a total of 5152 images in 11 categories.

Further sources for the dataset were the search engines **Bing and Google**. To download the Google images, the Chrome extension “Image Downloader” was used to acquire all usable images from the Google Image search with a minimum resolution of 120 × 120 pixels. Subsequently, the images were assigned to the respective class and manually reviewed. Images with more than one tool were split by cutting out the individual tools and saving them within separate instances. As for the ALET dataset, these images were further processed by resizing them to a square size with fill data and then scaling them to the defined size. The images from the Bing search engine were downloaded using a custom script. The further processing steps did not differ from the Google images. In total, these two sources provided 5447 images in 11 categories, of which 4194 images originated from the Google search engine and 1253 from the Bing search engine.

**Figure A5:**
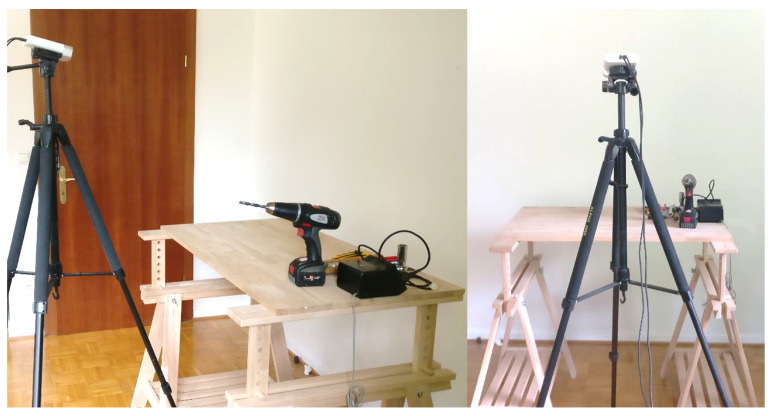
Test stand for capturing new images.

In addition to the image sources described above, **newly captured images** of tools in use were created with the Azure Kinect. The additional images were taken to increase the size of the dataset and especially to expand it with images of tools held in the hand. For this purpose, the images were taken at a test stand, as shown in Figure A5, with three varying camera angles to avoid a perspective bias within this dataset part. Due to missing hardware, no independent images could be generated for the tool class “riveter”. Some tools, such as pliers, were very different in appearance, whereas tools such as hammers had a similar external appearance. In order to reflect this visual variance in the dataset, multiple models for some of the tools were included. The tools used and the respective number of models are shown in Table A1. After acquisition, the images were sorted and scaled to the image classification size, just as for the other sources. This part of the dataset yielded a total of 11,304 images in 11 categories.

For the later experiments, the dataset was split into training, validation, and test data. According to [39], a distribution of 70% data for training, 15% for validation, and 15% for testing is recommended for medium-sized datasets. Thus, this partitioning of the dataset was applied for the later experiments.

#### 3.3.2. Image Classification Algorithm—Network Used

With several AI-based architectures available, we aimed to display the capability of our approach toward different frameworks and classification models. End-to-end object-detection algorithms, as used by the related work, i.e., YOLO models, are not necessary for this approach. Thus, only classification models were considered further.

In principle, any architecture that can be trained with the above-proposed database and is capable of handling multi-class classifications of the necessary above-derived complexity can be utilized in the proposed pipeline of Figure 4. To demonstrate the applicability with inherently different architecture types, we propose and tested the use of two different Neural Network types, which are typically put to use for such image classification tasks:

1. **DCNN-based classification** uses convolutional layers to extract features from given input images by convolving them with filters/kernels of various sizes. This allows the network to learn hierarchical representations of the input data that are increasingly abstract and complex. This principle has become state-of-the-art in various image-processing tasks and is well established with networks such as VGG [40], ResNet [41], MobileNet [42], and InceptionNet [43]. Out of these, ResNet50 was chosen as an exemplary model for DCNN-based classification principles due to its unique architecture, which allows a certain depth of the network without resulting in the vanishing gradient problem. The deep architecture of ResNet allows it to learn and capture intricate and abstract features from the input data. This capability enables ResNet to discriminate between subtle differences and fine-grained details, which may be crucial for distinguishing similar objects. We presumed the to-be-classified objects to be similar, and similar objects may have subtle variations in shape, texture, or appearance, which require a model to extract highly detailed features for accurate differentiation. The increased depth of ResNet enables it to learn and represent these subtle differences by building hierarchical and increasingly abstract representations of the input data.
2. **Transformer-based classification**, on the other hand, relies on a self-attention mechanism to encode the patches of the given input image and capture long-range dependencies between the different elements of the sequence. This allows the model to incorporate differentiated features in its final decision-making. With established success in natural language processing, the use of transformer networks for image processing is rising in popularity. Architectures such as Vision Transformer (ViT) [44] have shown initial success in outperforming DCNN-based networks in various applications and are chosen as an alternative to the ResNet model.

To demonstrate the feasibility of the approach with the different frameworks, we chose to implement the ResNet model through the Keras Applications API [45] and Tensorflow [46], whereas the Vision Transformer was implemented through the HuggingFace Transformers library [47]. In both instances, on the ImageNet [27] database, pre-trained models were used. Since the ImageNet pre-trained ResNet requires modifications before it can be fine-tuned to individual use cases, we propose replacing the head of the ResNet with an individual head as shown in Table 1, whereas the last dense layer of the network corresponds the number of tool classes. Furthermore, since the ResNet model requires modification before it can be used for fine-tuned applications, we propose to conduct a hyperparameter search for the parameters of the other added layers. The proposed search space is shown alongside Table 1.

The Vision Transformer network is not as heavily modified as the ResNet50. Thus, the necessary hyperparameter search was not as extensive and was limited to the training parameters’ learning rate, epochs, and batch size. The corresponding search spaces are listed in Table 2.

### 3.4. Experiments’ Description

To test the above-derived pipeline, different experiments were conducted. First, the experiments are described below, and in Section 4, the results are discussed in detail:

1. First, an **offline test** was conducted to display the capability of the above-outlined tool classification network as a stand-alone application. The two model architectures were trained with the training dataset and evaluated against the test dataset, introduced in Section 3.3.1.
2. Afterwards, experiments with different **live scenarios** were conducted to demonstrate and test the capability of the entire tool-recognition pipeline in combination with the action recognition. For this, several scenarios are defined:
  (a) **All classes**: This experiment was performed to test the principal applicability of the integrated image classificator in the pipeline. For this, all eleven tool classes and the none class were included in the test.
  (b) **All classes—challenging backgrounds**: To test the robustness of the approach in challenging environments, this scenario took place in front of chaotic backgrounds that pose distractions for typical vision applications. Backgrounds are considered challenging with multiple objects in view and/or complex shadow and light situations. This test was also performed with all classes.
  (c) **Three known classes—unknown instances of tools**: To test the transferability of the approach towards different instances of a tool class, additional tools of the three classes (1) pliers, (2) screwdriver, and (3) wrench were used. Those new instances distinguish themselves from the instances represented in the training dataset by different features such as color schemes and geometric individualities.
  (d) **Specific assembly scenarios**
    - One tool class and the none class: Assuming prior knowledge about a specific assembly step and anticipation of that step, a tool-recognition scenario would not include a manifold of tools in that step, but rather a single tool that was either used for that specific assembly step or not. Thus, a binary classification can take place between the none class and the specific tool class. To test the behavior of the AI detector between those two variants, this experiment was conducted.
    - Two tool classes: Similar to the above case, now, instead of the none class, another tool class was used in this test to demonstrate the increasing feasibility of the approach for reduced task complexities.

## 4. Results

**Figure 7:**
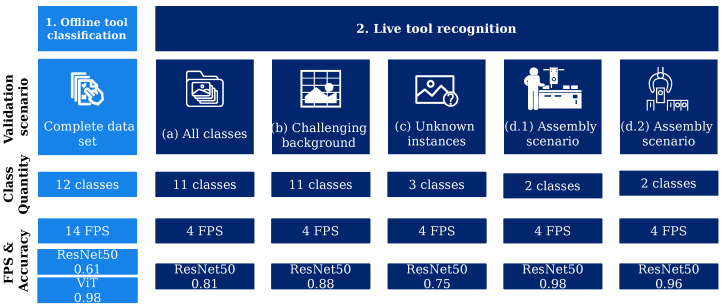
Overview of the conducted experiments and their results.

This section is divided into the results of the hyperparameter search for the chosen classification algorithms (ResNet 50 and Vision Transformer) and the results of the experiments described in Section 3.4. Figure 7 gives an overview of the experiments conducted and their results. Here, mainly the differences in the number of classes, as well as the core results regarding classification accuracies and achievable FPS are presented. Further result metrics are detailed in the corresponding subsections.

### 4.1. Hyperparameter Settings and Training

The proposed hyperparameter search for the ResNet50 model (see Table 1) was performed using a grid search algorithm. The resulting best model configuration is shown in Table 3.

The hyperparameter search for the Vision Transformer was performed using the Optuna HP Searcher [48]. The results are shown in Table 4.

### 4.2. Classification Results—Offline Test Data

**Figure 8:**
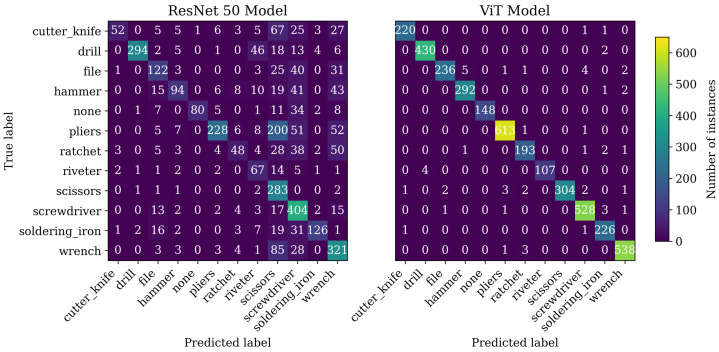
Confusion matrix of the classification test for both the ResNet50 model and the ViT model.

Both architecture types were trained according to their hyperparameter setting, as shown earlier, and were tested against the test dataset as introduced in Section 3.3.1. To display the classification results, both confusion matrices are shown in Figure 8. The classification accuracies for each model over all classes were:
**ResNet50**0.61**ViT**0.98

As further evaluation metrics, the sensitivity and the precision for each class were used. Sensitivity refers to the true positive rate, sometimes also referred to as recall. It measures the proportion of positive instances that are correctly identified by the model. A high sensitivity score indicates that the model identifies most of the positive instances correctly and has a low false negative rate. Precision indicates the proportion of true positive predictions and overall positive predictions. Thus, it describes a reference of true positives to the sum of True Positives (TPs) and False Positives (FPs). A high precision in a multiclass classification problem indicates that the model creates accurate positive predictions for a specific class.

Although the Vision Transformer’s accuracy significantly outperformed the ResNet model, the confusion matrix indicated that this performance loss was not equally distributed over all classes. Instead, it can be seen that nearly all classes had significant false positive values for the three classes scissors, screwdriver, and wrench.

The results for the class “scissors” were particularly noticeable. The calculated sensitivities for this class displayed the highest sensitivity result compared to other classes, but the precision value was the lowest of all values. This indicated that a classification of “scissors” was very often scissors. However, at the same time, other classes were often classified as “scissors”. The results of the class “cutter knife” were interpreted as the opposite of this. Here, the sensitivity was very low, with a relatively high positive predictive value at the same time. According to this, the images of the class “cutter knife” were often classified as other classes. The other classes, however, were almost never classified as “cutter knives”.

#### Reducing the FP Quota of the ResNet

To alleviate the tendency of ResNet to produce false positives, we performed an experiment that took the network’s confidence into account. Three different confidence thresholds were applied to the evaluation. Predictions that were below the threshold were not considered. Although this approach significantly increased the false negative rate, it might be useful for such use cases where false positives might do harm in follow-up processing steps.

Using this approach, the increased accuracy results were gained as given in Table 5.

While the accuracy for the whole dataset was 61.5%, introducing the confidence threshold increased the accuracy significantly. If a threshold of 10% was adopted, the accuracy improved by 3.7%. The change in accuracy was even more significant when a threshold of 17% was adopted. This improved the accuracy to 88%. Here, it is reasonable to assume that higher recognition confidence led to better classification results. A threshold of 17% for detection confidence also strongly affected the sensitivity and positive predictive value of all classes. In this case, the positive predictive value was above 74% for all classes. The sensitivity could also be improved for all classes. Only the two classes “File” and “Ratchet”, with, respectively, 54.5% and 30%, had sensitivities below 69% when the detection confidence threshold was set to 17%. By introducing a threshold value of 17%, the sensitivity of the class “cutter knife” could be improved from 26.1% to 69.7%. The class “None” also benefited from such a value. Thus, the sensitivity and the positive predictive value could be improved significantly. Both values reached 100% if a threshold of detection confidence of 17% was chosen. The integration of a threshold for this criterion, therefore, seemed to have a significant impact on the accuracy of the classification results and, thus, represented a possible improvement approach for the overall process.

### 4.3. Results of Live Scenarios—Online Pipeline

An image-processing pipeline, as presented in Section 3, was implemented using the Python API for the Azure Kinect SDK and the Tensorflow ML framework [46]. The physical setup is shown in Figure A5. A Windows Environment with an Nvidia GTX 1050 was used as a processing system. Due to the computational limitations of this live setup, only the ResNet network could be applied in the following scenarios. To achieve comparability between the following experiments, the same light situations were created, and the same human actor with the same clothing performed the experiments. Due to the limited availability of riveter hardware, no exhaustive experiments were possible with this tool class. Thus, the following experiments considered a reduced dataset of up to ten tool classes plus the none class.

#### 4.3.1. All Classes—Normal and Challenging Backgrounds

The first experiment conducted aimed to demonstrate the principle feasibility of the entire pipeline with multiple classes. Hence, the first approach used simple uniform backgrounds. The second approach then introduced modified, more chaotic backgrounds.

The results of the sensitivity and precision metrics for each class are indicated in Table 6. The difference between normal and modified backgrounds is also shown for each class and metric. In general, the classification accuracy for the modified background experiment was higher than that of the normal background:
**Normal**0.81**Modified BG**0.88

The sensitivity for the class “file” on the modified background was 0, resulting in an incalculable precision for this class with a divisor being 0.

#### 4.3.2. Three Known Classes—Unknown Instances

**Figure 9:**
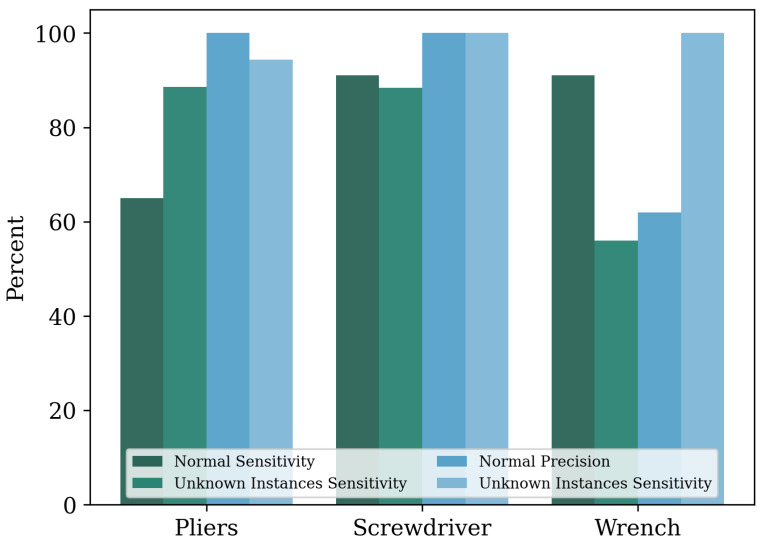
Comparison of sensitivity and positive predictive value for untrained instances of known classes.

To test whether the dataset enabled the tool-recognition classificator to be transferred to new instances of already known classes, new instances for three classes were tested. Figure 9 displays the sensitivity and precision for the previously used tools and the new unknown instances of tools.

#### 4.3.3. Two Classes—Assembly Scenarios

To test the behavior of the system with only two classes to distinguish, two different scenarios were chosen. First, the class “Screwdriver” was combined with the class “None”, and second, the classes “Drill” and “Screwdriver” were combined for a scenario. The classification accuracies reached for the two scenarios are:
Screwdriver and none98.3%Drill and screwdriver96.5%

#### 4.3.4. Measuring Online Usability of Toolbox—FPS

To measure the online usability of the tool-recognition pipeline, its frame rate was investigated. First, the frame rate running on the hardware as described in Section 4.3 was measured, resulting in an average frame rate of

4 FPS.

To determine the bottleneck of the system, the trained ResNet model was deployed to the ONNX format and evaluated on a system with an Nvidia RTX3090 GPU, resulting in

14 FPS.

## 5. Discussion

In this section, the developed method and the results obtained in the experiments are discussed. First, the offline image classification and then the results of the online pipeline experiments are addressed. Furthermore, the correlation between the computational effort and the hardware used, along with the resulting frame rate is discussed. Besides the experiments, the bias of the dataset is addressed and discussed. Finally, the approach is evaluated against the considered related work and the overall goal of this work.

### 5.1. Discussion of the Offline Image Classification Results

The significant difference in performance between the ResNet model and the Transformer model clearly indicated the superiority of the latter for this use case. This performance was primarily due to the misclassification of the tools into the “scissors”, “screwdriver”, and “wrench” classes. The best-classified class was “Drill”. Therefore, images with this class were mostly classified as such. One reason for this may be that it is a very large tool, which also has a clearly recognizable and very individual shape.

**Figure 10:**
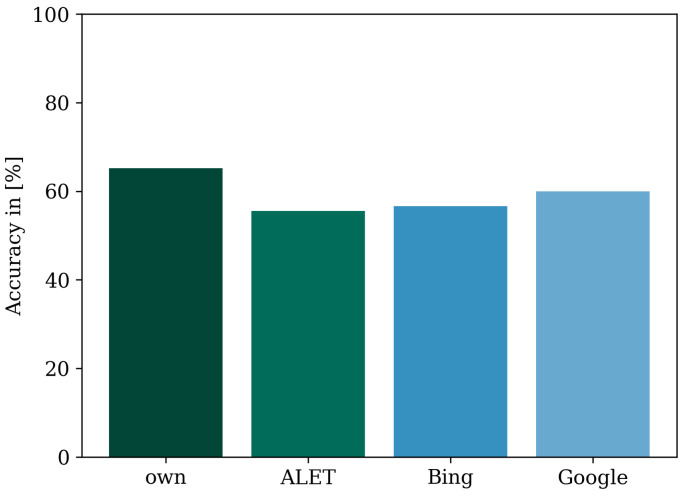
Accuracy of the offline classification models shown for each image data source.

To further investigate this behavior, we differentiated the classified images according to the image source. It can be seen that the accuracies lied between the values 65.2% and 55.5% (see Figure 10). Since our own images dominated the whole dataset and thus also in the training, validation, and test datasets, it can be assumed that this affected the validation results. The image source of the search engine Bing was the least represented and was only recognized with an accuracy of 56.6%. The image source with the lowest accuracy value was the ALET dataset with 55.5%. This may be due to the poor image quality. Overall, it can be concluded that, as the proportion of images from an image source increased, the accuracy achieved relative to the image source also increased.

For the evaluation of the image sources, it should also be noted that a non-negligible pre-processing effort was made. Thus, the images were extracted from the image sources of the ALET dataset using the coordinates provided by the publishers. Images from the Bing and Google search engine image sources were extracted manually. Additionally, all images were manually sifted, and those that were deemed unidentifiable were sorted out. Accordingly, it cannot be ruled out that different results were obtained with a different data preparation of the same data.

### 5.2. Discussion of the Online Pipeline Experiments

Evaluating the online experiments, no significant correlation was found between the well- and reliably classified classes and the number of instances in the dataset. While the classes “Drill” and “Screwdriver” had more than 2500 instances, the classes “cutter” and “soldering iron” had less than 1500 instances. They, thus, tended to be among the less-frequently represented classes. The class with the most instances, “Pliers”, had the best precision, but at the same time, only a medium sensitivity value. Two classes that had a similar number of instances in the dataset were “screwdriver” and “wrench”. While the sensitivity of the two classes was almost identical, the precision values differed by 36.5% points. Therefore, it is reasonable to assume that the number of instances was less important than the quality and appearance of the tool. However, it can be seen from the class “pliers” that a positive prediction value of 100% can only be achieved with a particularly large number of instances. Considering all classes, no direct correlation can be established between the number of images of the respective class in the dataset and the resulting performance in these tests. Furthermore, it can be concluded that, for tools with high optical variance (i.e., many different features, e.g., pliers), the dataset needs to reflect this optical variance and, therefore, requires a high number of instances. As a result, some classes may require significantly more instances than tools with a low optical variance to achieve the same performance. The expected decrease of the metric values for classification with challenging background conditions did not take place. This can be explained by the fact that a large part of the background was not visible in the images used for classification due to the ROI. Accordingly, an influence of the background when using an ROI in conjunction with a camera position of about 60° was less of a problem than might have been suspected. The poor values for the classification rates of the class “file” in the online pipeline may also be due to the generally difficult classification of this class, as already discussed.

Reflecting on the test with unknown instances of known classes, it can be inferred that the network might be capable of handling such new instances. However, it can be seen that the class “wrench” yielded a significantly worse sensitivity (56%). Instead of the class “wrench”, the classes “scissors”, “cutter knife”, and “pliers” were increasingly detected. In contrast, the performance of the remaining tools can be rated sufficient. The result was surprising in that wrenches appear quite similar from a human perspective. A deterioration of the classification performance due to high optical variance was not notable. However, it is precisely this low optical variance that could lead to worse performance in future applications. Thus, it cannot be excluded that the features of the wrench instances within the training dataset contained several common features that were not shared by new instances. This might indicate a bias in the network towards wrenches that contain a certain set of features.

As expected, the accuracies for the assembly situation scenarios with only two classes performed significantly better. The decrease in classification complexity generally benefited the performance. Thus, for industrial use cases, prior knowledge of the tools to be handled enabled the utilization of a specifically trained recognition algorithm with reduced complexity, which can significantly boost the accuracy of the herein presented approach.

### 5.3. Discussion of the Resulting Frame Rate

As presented in Section 4.3.4, the resulting frame rate of just above 4FPS did not meet the initial requirement of 30 FPS for a near-real-time live prediction. Such frame rates might be sufficient for slow assembly processes, but they are not suitable for efficient human–machine collaboration. One reason for this behavior could be that the image classification network used was too complex to achieve 30 FPS with the given hardware. A less-complex model could improve this, but it might also have a negative impact on the accuracy of the classification results. Additionally, it is possible that the specific combination of the camera with the hardware used did not support video data containing skeletal information at 30 FPS. If this is the case, reducing the skeletal data to the relevant joints could improve the result. For the classification, the software API or library CUDA, respectively cuDNN, were used to ensure or improve the integration of the GPU. However, it cannot be determined with conclusive certainty whether this improvement potential is fully exploited in the software–hardware combination used. Thus, it cannot be excluded that a better classification speed would be achievable if the mentioned software packages were used optimally. In addition, other hardware components, especially GPUs and CPUs, could contribute to improving the classification speed. Moreover, the processing overhead of tool recognition without classification seems significant. The image classification as a stand-alone module achieved a classification speed of 14 FPS, while the tool recognition could only achieve 4.1 FPS. Therefore, in addition to improving the image classification, the processing steps of the tool recognition should be improved with respect to speed. For this purpose, experiments should be carried out to determine which aspects of the preceding steps (detection and transmission of video and skeleton information, generation of ROIs, cropping, and post-processing of the extracted image) are responsible for this reduction.

### 5.4. Discussion of Bias in the Dataset

With the live experiments being performed by a single actor, the transferability of the trained models and the acquired data to other users might be challenging. As mentioned above, data scraping and pre-processing are mostly conducted by a single person. Comparing this against the work of [49] led to the conclusion that the used dataset in this work most likely contained bias. However, using different search machines and combining the different datasets mitigated this bias to some extent, but as shown by Figure 10, not in its entirety. Although the findings of the work were not fundamentally challenged by this probable bias, transfer and re-use of the utilized data and the models should be performed after careful consideration of possible bias.

### 5.5. Comparison of the Approach to Related Work

**Figure 11:**
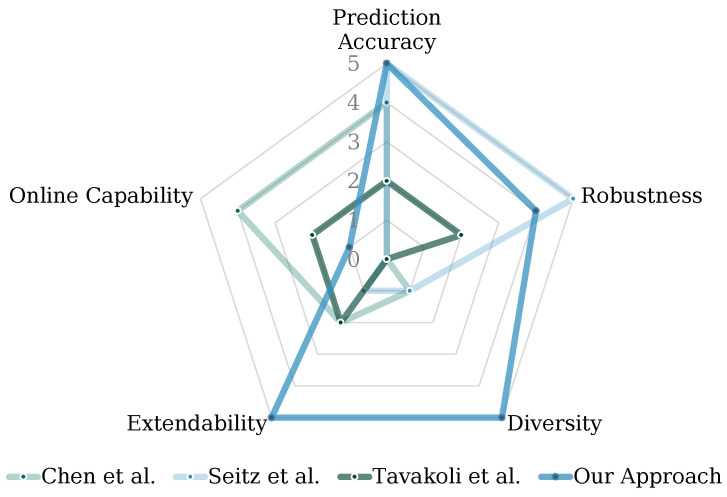
Comparison of our approach to the considered related work [9,25,26] under consideration of the five criteria of online capability, prediction accuracy, robustness, diversity, and extendability.

The discussion of existing approaches in the field of tool recognition in manual assembly situations utilized the above-introduced evaluation criteria. The related work showed the principle applicability of visual approaches for tool recognition, considering challenges such as occlusion. The reviewed approaches, such as those in [9,25,26], demonstrated different strengths and limitations in terms of tool diversity, robustness, and online capability. Our presented framework aimed to address these limitations and provide a solution that combines high prediction accuracy, robustness, tool diversity, and online capability while allowing for extension and flexibility in handling new components, processes, or tools. With respect to the desired frame rate, our approach fell short of [9]. One of the primary criteria focused on in this work was “Robustness”. In the developed concept, not only tool classes, but also a “no tool” class were implemented, improving our robustness in comparison to other approaches. This allowed the approach to be used for assembly steps containing tools, as well as without tools. Our accuracy can be considered competitive with the other approaches, with up to a 98% accuracy achieved. Furthermore, our approach surpassed other work with respect to the approach’s extendability, as well as its considered diversity. In summary (see Figure 11), our approach extended the state-of-the-art in two main aspects: diversity and extendability, while maintaining high prediction accuracy, as well as robustness. However, the shortcoming of the online capability has to be addressed in future approaches.

### 5.6. Discussion of the Assembly Step Estimation

Since this work aimed to enhance HAR methods for efficient ASE in manual and multi-variant assembly, this objective was evaluated by the following. As implied in Section 2, an online tool recognition can gain valuable information, improving the estimation of the assembly progress. This approach offers a method for such an online tool recognition considering the requirements from HAR. The developed and tested system was capable of recognizing tools in use and distinguishing them from tools not used by tracking the human worker with an auspicious accuracy. Despite open issues such as low frame rates due to hardware limitations, this work provides an important contribution to the improvement of HAR for ASE. Within the scope of this work, the proposed building block of tool recognition in [11] was investigated and developed and can, thus, be integrated into the overall concept for ASE.

## 6. Conclusions and Future Work

In conclusion, this paper implemented a novel approach to enhance the recognition of human actions in manual assembly by introducing a tool-recognition pipeline. The proposed online-capable approach included an adaptive ROI for the bounding box using pose estimation of the human worker. The approach demonstrated high accuracy in recognizing handheld tools in manual assembly processes. The generated dataset contained a sufficient amount of images for training, validation, and testing, and the study demonstrated the general applicability of different classification algorithms for this type of task.

The discussion of the results conducted in this paper revealed that the proposed approach performed well, achieving high accuracies in tool recognition. However, the achieved frame rate of the system was lower than anticipated and did not suffice for some sophisticated real-world applications. The introduction of tool recognition as an additional feature provided valuable information for detecting human actions in manual assembly. Nonetheless, there were some limitations and areas for improvement, such as the need for further optimization of the algorithm and the possibility of bias in the generated dataset.

In summary, this paper provided insights into the use of tool recognition to enhance human action recognition and demonstrated the potential of the proposed approach for future research. The evaluation of the results highlighted both the strengths and limitations of this approach and suggested avenues for further development of the tool-recognition pipeline, such as optimizing the system’s frame rate to increase its usability for more sophisticated real-world applications.

## Figures and Tables

**Table 1:** Proposed modification of the ResNet 50 and the corresponding hyperparameter search space.

Layer	Hyperparameter	Search Space
ResNet50 Base Model	Trainable Layers	[0…197]
Flatten	-	-
Dense	Number of Neurons	[50…5000]
	Activation Function	[Relu, tanh]
Dropout	Option	[True, False]
	Dropout Rate	[0.1…0.2]
	Optimizer	[Adam, SGD, RMSPROP, AdaDelta]
	Learning Rate	[$1\times 10^{-4}$ …$1\times 10^{-7}$]
	Batch Size	32
	Epochs	25

**Table 2:** Hyperparameter search space for the Vision Transformer.

Hyperparameter	Search Space
Epochs	[1, 5, 10, 15]
Learning Rate	[$1\times 10^{-6}$…$1\times 10^{-4}$]
Batch Size	[8, 16, 32, 64, 128]

**Table 3:** Result of hyperparameter study for ResNet 50.

Layer	Hyperparameter	Best Trial
ResNet50 Base Model	Not Trainable Layers	0–12
ResNet50 Base Model	Trainable Layers	120–197
Dense	Number of Neurons	3296
	Activation Function	Relu
Dropout	Option	True
	Dropout Rate	0.10
	Optimizer	RMSprop
	Learning Rate	$1.78\times 10^{7}$
	Epochs	55
	Batch Size	32

**Table 4:** Result of hyperparameter study for the Vision Transformer.

Hyperparameter	Best Trial
Number of Trials	50
Epochs	10
Learning Rate	$6.51\times 10^{-5}$
Batch Size	32

**Table 5:** Achieved accuracies of ResNet50 using confidence thresholds.

Confidence Threshold	Accuracy
10%	0.65
15%	0.84
17%	0.88

**Table 6:** Sensitivity and precision results for each class of the live prediction online scenario for both the normal background experiment and the Modified Background (Mod. BG) experiment.

	Sensitivity			Precision		
Class	Normal	Mod. BG	Delta	Normal	Mod. BG	Delta
Cutter	99.2	100.0	−0.8	99.7	100.0	−0.3
Drill	100.0	100.0	0.0	85.3	79.1	6.2
File	32.6	0.0	32.6	62.8	-	-
Hammer	51.5	89.6	−38.0	99.6	100.0	−0.4
None	82.8	97.8	−15.0	60.4	100.0	−39.6
Pliers	65.6	76.4	−10.8	100.0	99.2	0.8
Ratchet	87.0	100.0	−13.0	82.9	78.4	4.5
Scissors	95.0	100.0	−5.0	78.8	52.2	26.6
Screwdriver	92.9	97.6	−4.8	99.7	100.0	−0.3
Soldering Iron	96.6	100.0	−3.4	98.7	100.0	−1.3
Wrench	92.8	84.8	8.0	63.2	100.0	−36.8

## Data Availability

Due to the usage of data with unclear licenses, only the generation of the dataset can be described in a traceable manner (see Section 3.3.1), but it cannot be made publicly available.

## Abbreviations

The following abbreviations are used in this manuscript:

HAR	Human Action Recognition
HMI	Human–Machine Interface
HRC	Human–Robot Collaboration
ASE	Assembly Step Estimation
HMM	Hidden Markov Model
FSM	Finite-State Machine
MTM	Methods-Time Measurement
ROI	Region of Interest
SDK	Software Development Kit
BB	Bounding Box
ViT	Vision Transformer
TP	True Positive
FP	False Positive

## Appendix A

**Table A1:** Model quantities for different tool classes.

Tool	Model Quantity
Scissors	3
Hammer	1
Soldering iron	1
Drill	2
Cutter Knife	1
Screwdriver	4
Wrench	4
Ratchet	2
File	2
Pliers	5
